# *In Vitro* Drug Response and Efflux Transporters Associated with Drug Resistance in Pediatric High Grade Glioma and Diffuse Intrinsic Pontine Glioma

**DOI:** 10.1371/journal.pone.0061512

**Published:** 2013-04-29

**Authors:** Susanna J. E. Veringa, Dennis Biesmans, Dannis G. van Vuurden, Marc H. A. Jansen, Laurine E. Wedekind, Ilona Horsman, Pieter Wesseling, William Peter Vandertop, David P. Noske, GertJan J. L. Kaspers, Esther Hulleman

**Affiliations:** 1 Department of Pediatric Oncology/Hematology, VU University Medical Center, Amsterdam, The Netherlands; 2 Department of Neuro-Oncology Research Group, VU University Medical Center, Amsterdam, The Netherlands; 3 Department of Clinical Genetics, VU University Medical Center, Amsterdam, The Netherlands; 4 Department of Pathology, VU University Medical Center, Amsterdam, The Netherlands; 5 Department of Neurosurgery, VU University Medical Center, Amsterdam, The Netherlands; 6 Department of Pathology, Radboud University Nijmegen Medical Centre, Nijmegen, The Netherlands; University of Chicago, United States of America

## Abstract

Pediatric high-grade gliomas (pHGG), including diffuse intrinsic pontine gliomas (DIPG), are the leading cause of cancer-related death in children. While it is clear that surgery (if possible), and radiotherapy are beneficial for treatment, the role of chemotherapy for these tumors is still unclear. Therefore, we performed an *in vitro* drug screen on primary glioma cells, including three DIPG cultures, to determine drug sensitivity of these tumours, without the possible confounding effect of insufficient drug delivery. This screen revealed a high *in vitro* cytotoxicity for melphalan, doxorubicine, mitoxantrone, and BCNU, and for the novel, targeted agents vandetanib and bortezomib in pHGG and DIPG cells. We subsequently determined the expression of the drug efflux transporters P-gp, BCRP1, and MRP1 in glioma cultures and their corresponding tumor tissues. Results indicate the presence of P-gp, MRP1 and BCRP1 in the tumor vasculature, and expression of MRP1 in the glioma cells themselves. Our results show that pediatric glioma and DIPG tumors *per se* are not resistant to chemotherapy. Treatment failure observed in clinical trials, may rather be contributed to the presence of drug efflux transporters that constitute a first line of drug resistance located at the blood-brain barrier or other resistance mechanism. As such, we suggest that alternative ways of drug delivery may offer new possibilities for the treatment of pediatric high-grade glioma patients, and DIPG in particular.

## Introduction

Pediatric high grade glioma (pHGG) constitutes 15–20% of pediatric central nervous system tumors [1]. These aggressive tumors are difficult to treat, and are associated with an extremely poor prognosis. The extent of surgical resection is the most important clinical prognostic factor in these patients [2]. Together with radiotherapy, which is a standard component of postoperative management, a 2-year-survival rate of 10–30% for supratentorial HGG has been established [1]. Diffuse intrinsic pontine glioma (DIPG), an infiltrative tumor typically originating in the pons, does not qualify for surgical resection due to its delicate location. In DIPG radiotherapy prolongs progression free survival (PFS) and improves quality of life, yet, the median overall survival (OS) in these children is still only nine months [3]. Unfortunately, no chemotherapeutical regimens or alternative radiation options have successfully improved OS or PFS in children with HGG and DIPG [4], [5]. These disappointing results emphasize the need to identify effective drugs. Therefore, we screened a heterogeneous group of primary pHGG cell cultures, including three DIPG cultures, for their sensitivity *in vitro* to different drugs.

To clarify whether a lack of clinical response results from tumor cell resistance or from poor drug delivery to the tumor cells, we also explored the mode of drug resistance in these tumors. In particular, we focused on one of the main mechanisms of drug resistance in the brain mediated by overexpression of ATP-binding cassette (ABC) transporters. Drug delivery to the brain is hampered by the presence of P-glycoprotein (P-gp, ABCB1), breast-cancer-resistance protein (BCRP, ABCG2), and multidrug-resistance-associated proteins (MRPs, ABCC1) [6]. Presence of these transporters on tumor cells or (peri)tumoral vasculature results in active efflux of chemotherapeutics by transmembrane transport, leading to a decrease in intracellular drug levels and subsequently a decrease in their cytotoxic activity [7]–[9].

Here we show that several classic chemotherapeutic drugs display a high cytotoxicity in primary pediatric glioma cell cultures *in vitro*, in physiologically relevant doses. Immunohistochemical staining on the corresponding tumor tissue indicates the presence of P-gp, MRP1, and BCRP1 on the tumor vasculature, while only MRP1 is also expressed in glioma cells. These findings suggest that the presence of all three major drug efflux pumps in the blood-brain-barrier (BBB) may form a first line of resistance of pHGG to treatment with classic chemotherapeutic drugs which by themselves are capable of inducing cytotoxicity in pediatric glioma cells. The drug transporters at the BBB impede drug delivery to the tumor site, while the presence of MRP1 protein in glioma cells themselves, may further protect the tumor cells from chemotherapy.

## Results

### Tumor and Patient Characteristics

Primary cell cultures were established from tumor material from nine children, aged one week to 11 years, diagnosed with high grade glioma (Table 1). In six patients, the tumor was located supratentorially, among these were five glioblastoma multiforme (GBMs), one anaplastic astrocytoma (AA), and one anaplastic oligodendroglioma. Among the GBMs was one rare case of giant cell GBM. Also, three DIPGs were obtained with different histological grading, including a GBM, an AA WHO grade III, and one diffuse fibrillary astrocytoma. In most cases resections were performed before initiation of therapy, except for one DIPG patient, from whom autopsy material was retrieved approximately two hours after death (VUMC-DIPG-01). Malignancy of the established cell lines was confirmed by determining the chromosomal aberrations, either by array CGH or by classical karyotyping. Apart from VUMC-HGG-06 and VUMC-HGG-07, all cell cultures showed abnormalities, varying from small deletions in VUMC-HGG-02 and -03, to extensive chromosomal changes in VUMC-DIPG-A. Within the VUMC-DIPG-A culture, diverse chromosomal aberrations were identified.

**Table 1:** Tumor and patient characteristics corresponding with the primary cell cultures.

VUMC-	Sex	Age	Histology	Location	Surgery	Radiotherapy	Chemotherapy	Survival	Classical karyotype	Deduced copynumber karyotype^73^ (array results)
	*f/m*	*years*				*Gy (cum.)*		*months*		
**HGG-01**	f	14	AA	thalamus	STR	54 Gy	CCNU, vincristine	15	−	-X,del(1)(pterp12),dup(4)(q12q12), −10, −11,del(12)(pterp11.21 del(12)(q15qter), −13, −15, del(16)(p11.2p11.2), −18, del(20)(pterp11.23)
**HGG-02**	f	0	GBM	right frontal lobe	STR	n.a.	n.a.	0	46,XX [10]	dup(10)(q11.2q12)
**HGG-03**	f	1	GBM	left hemi-sphere	GTR	−	valproïc acid	6	46,XX [20]	del(10)(q11.2q12)
**HGG-05**	f	11	GC GBM	right frontal lobe	STR	60 Gy	vincristin, carboplatin, cyclo- phosphamide, etoposide	12	46,XX,i(17) (q10) or add(17) (p?) [2]/40,XX, −6, −13, add(18)(p11,?2), −19, −20, 21,add(21) (p11.2) [1]/46,XX [16]	arr(1–22,X)x2
**HGG-06**	f	5	AO	left hemi-sphere	STR	54 Gy	procarbazine, CCNU, vincristine	84**	46,XX [13]	arr(1–22,X)x2
**HGG-07**	f	8	GBM	thalamus	GTR	−	CCNU, vincristine, prednisone	24	46,XX [10]	arr(1–22,X)x2
**DIPG-01**	m	5	GBM	pons	autopsy	45 Gy*	temozolomide	9	46,XY,del(4)(p12) [3]	−
**DIPG-A**	f	4	AA	ventral pons	PR	54 Gy	−	8	44,XX,del(1) (q21qter), dup(7) (pterp12.3), del(7)(q31.32q31.32), del(17) (pterp11.2), −18, −21	40∼44,XX, −4 [2], −5 [2], −6 [3],+7 [3], −13 [3], −15 [2], −17 [2], −18 [5], −21 [4], add(21)(p11.2) [2], −22 [3],+1∼7mar[cp6]/79∼84,XXXX, −4, −5, −5,der(5)t(5;8)(q35;q10) [1], −6, −6, −8, −8, −11,add(11)(p11.2) [1], −13, −13,add(13)(q34) [1], −14, −16, −17, −17, −18, −18, −21, −21, −21, −22, −22, 6∼17mar[cp3]
**DIPG-B**	f	3	DA2	pons	PR	−	carboplatin, procarbazine, cisplatin, Etoposide, vincristine, cyclophosphamide	18	46,XX+der(1)t(1;21) (q10;q10), −21 [1]/45,XX, +del(1)(p?13), −3,+8, −10,+21 [1]/46,XX, −?8, +?10,+?15, −?17 [1]	dup(1)(q12qter)

f: female, m: male, AA: anaplastic astrocytoma, GBM; glioblastoma, GC GBM: giant cell glioblastoma multiforme, AO: anaplastic oligodendroglioma, DA2: diffuse fibrillary astrocytoma, GTR: gross total resection, STR: subtotal resection, PR: partial resection. Chemotherapy was administered adjuvant to resection and/or radiotherapy in all treated patients.

* hypofractionated radiotherapy 15 fractions of 3 Gy,

** survivor, n.a.: does not apply.

### Chemosensitivity of Primary Pediatric High Grade Glioma Cultures

**Figure 1:**
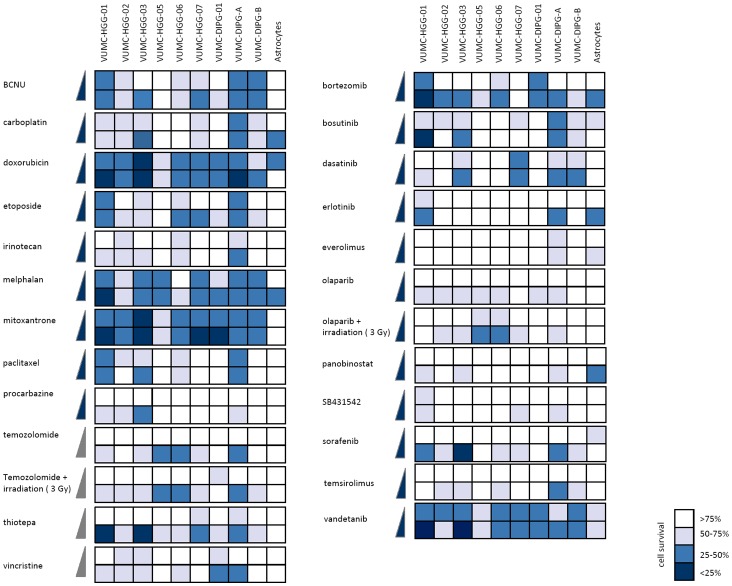
a. Cell survival among primary pHGG cultures exposed to classical chemotherapeutic drugs. b. Cell survival among primary pHGG cultures exposed to novel drugs.

In order to determine the sensitivity of primary pHGG cultures to a series of drugs, a small chemical screen was designed, which included conventional chemotherapeutic drugs of a variety of classes, (anthracyclins, alkylating agents, topoisomerase I/II inhibitors, nitrosureas, mitotic inhibitors, anti-metabolites), and small molecule inhibitors targeting specific proteins that are often upregulated in pHGG or DIPG (Figure S1, Material and Methods S1). Drug concentrations used were based on IC_50_ values reported for adult malignant glioma cell lines (Table 2 and 3). The effect of the compounds on cell survival was tested at least four times for each primary culture. Robustness of the assay was assessed by calculating the coëfficient of variation (CV = 9.4±3.7) and the Z’ factor for each cell line. Except for VUMC-DIPG-01, all Z’ factors indicated a powerful assay (Z’ = 0.6±0.09). In addition, drug sensitivity of primary human astrocytes was measured, to determine the therapeutic window for the various drugs. As shown in Figure 1a, cell cultures VUMC-HGG-01, and VUMC-DIPG-A were relatively sensitive, with <50% cell survival in response to more than half of the chemotherapeutics. In contrast, VUMC-HGG-05 was the most resistant cell line of the panel, remaining unaffected by the majority of compounds. BrdU incorporation studies showed that this resistance or sensitivity was not correlated to proliferation index (Figure S2, Material and Methods S1). The only drug capable of inducing significant toxicity in VUMC-HGG-05 was melphalan. In general, melphalan, the anthracyclines doxorubicine and mitoxantrone, and BCNU most effectively affected cell survival, followed by etoposide, thiotepa, and carboplatin. Among the novel drugs, bortezomib had a significant anti-glioma effect; in six out of nine cultures, exposure to 100 nM bortezomib resulted in >50% reduction in cell survival. Bosutinib, dasatinib, sorafenib, and olaparib (either as monotherapy or in combination with irradiation), had an effect in approximately 4–5 cell cultures. In VUMC-HGG-05, VUMC-HGG-06, and VUMC-HGG-07 the effect of olaparib increased when combined with irradiation. Although the drug dose ranges in our study overlapped with published IC_50_ values in cancer cell lines, no or little effect was observed with erlotinib, everolimus, panobinostat, and SB431542 (Figure 1b).

**Table 2:** Classical drugs used in this study.

Drugs	Mechanism of action	Substratefor MRP1	Substratefor P-gp	Substratefor BCRP	Concentrations usedin this study	Published in vitro IC^50^ inmalignant glioma	Published peak plasma concentration in children
**BCNU**	Alkylating agent	+	−	−	100 uM, 1 mM	37 uM [43], 173 uM [44], 467 uM [45]	not reported
**Carboplatin**	Alkylating agent	+	+	−	260 uM, 2.6 mM	80 uM [46]	155 uM (57.6 ug/mL) [47]
**Doxorubicin**	Topoisomerase II	+	+	+	100 nM, 1 uM	500 nM [48]	470 nM (273 ug/L) [49]
**Etoposide**	Topoisomerase II	+	+	+	20 uM, 200 uM	8.5 uM [50]	31 uM (18.5 ug/mL) [51]
**Irinotecan**	Topoisomerase I	+	+	+	1 uM, 10 uM	not reported	89 uM [52]
**Melphalan**	Alkylating agent	−	+	−	40 uM, 400 uM	35.4–103 uM [53]	not reported
**Mitoxantrone**	Topoisomerase II	+	+	+	100 nM, 1 uM	230 nM [54]	not reported
**Paclitaxel**	Microtubuli	+	+	−	10 nM, 100 nm	81 nM [55]	8.3–38.9 uM [56]
**Procarbazine**	Alkylating agent	+	−	−	500 uM, 5 mM	not reported	not reported
**Temozolomide**	Alkylating agent	−	+	+	50 uM, 500 uM	71 uM [57], 258 uM [58]	75 uM (14.6 mg/L) [59]
**Thiotepa**	Alkylating agent	−	+	−	25 uM, 250 uM	not reported	13 uM [60]
**Vincristine**	Microtubuli	+	+	−	10 nM, 100 nM	81 nM [61]	4–38 nM (1–32.1 ug/L) [62]

Mechanism of action, concentrations, and ABC efflux transporter substrate specificity for classical therapeutics.

**Table 3:** Novel drugs used in this study.

Drugs	Target	Substrate for MRP1	Substrate for P-gp	Substrate for BCRP	Concentrations used in this study	Published *in vitro* IC_50_ in human cancer cell lines
**Bortezomib**	26S proteasome	−	+	−	10 nM, 100 nM	28.9–48.2 nM (glioma) [63]
**Bosutinib**	c-Abl, Src, HDAC	+	+	−	1 uM, 10 uM	1.3 (colorecca) [64], 5.7 uM (melanoma) [65]
**Dasatinib**	BCR/ABL, c-Kit, Src, Ephrin	+	+	+	100 nM, 1 uM	210 nM–1.5 uM (glioma) [66]
**Erlotinib**	EGFR	+	+	+	5 uM, 50 uM	9 uM (glioma) [67]
**Everolimus**	mTOR	−	+	−	100 nM, 1 uM	271 nM (glioma) [68]
**Olaparib**	PARP	−	+	+	1 uM, 10 uM	1.42–7.43 uM (glioma) [69]
**Panobinostat**	HDAC	−	+	+	50 nM, 500 nM	not reported
**SB431542**	TGFß, Alk	−	+	+	1 uM, 10 uM	0.1–10 uM (dose range used in *in vitro* studies) [70]
**Sorafenib**	Raf, PDGFR, VEGFR, c-Kit	−	+	+	2.5 uM, 25 uM	5–20 uM (glioma) [71]
**Temsirolimus**	mTOR	−	+	−	1 uM, 10 uM	1.4 uM (variety) [72]
**Vandetanib**	EGFR, VEGFR, Ret	−	−	+	10 uM, 100 uM	10 uM [73]

Drug targets, concentrations, and ABC efflux transporter substrate specificity for novel, targeted drugs.

### Expression of Drug Efflux Transporters in pHGG

**Figure 2:**
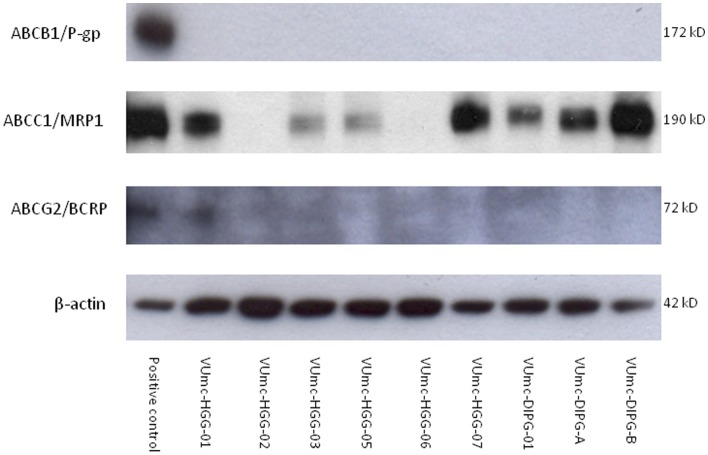
Western blot for detection of P-gp, MRP1, and BCRP1 in pHGG cultures. MW represents approximate molecular weight of these proteins, as indicated at the right. The pHGG lanes were loaded with 20 µg of protein, the lanes with positive controls were loaded with 5 µg of protein.

In order to determine whether ABC transporters play a role in the responses of our cell cultures, we assessed the presence of the main ABC transporter proteins P-gp (ABCB1), MRP1 (ABCC1) and BCRP1 (ABCG2). Therefore, Western blotting experiments were performed on the pHGG cell cultures (Figure 2). MRP1 was detected in seven out of nine primary glioma cultures, with a variable intensity (2 low, 5 high expression). High MRP1-expression was detected in VUMC-HGG-01, VUMC-HGG-07, VUMC-DIPG-A, and VUMC-DIPG-B. All cultures were negative for P-gp, and in all except VUMC-HGG-01, BCRP1 was also absent. The MCF7/P-gp and MCF/BCRP1 cell lines were used as a positive control for P-gp, and BCRP1 [10], [11], and the 2008/MRP1 cell line for MRP1 [12]. Several bands on the BCRP1 blot appeared at a molecular weight which was higher than expected (220 kD instead of 72 kD) (data not shown). These bands were present in the cell lysates of all glioma cultures, and were of pronounced intensity in the six supratentorial pHGG cultures.

**Figure 3:**
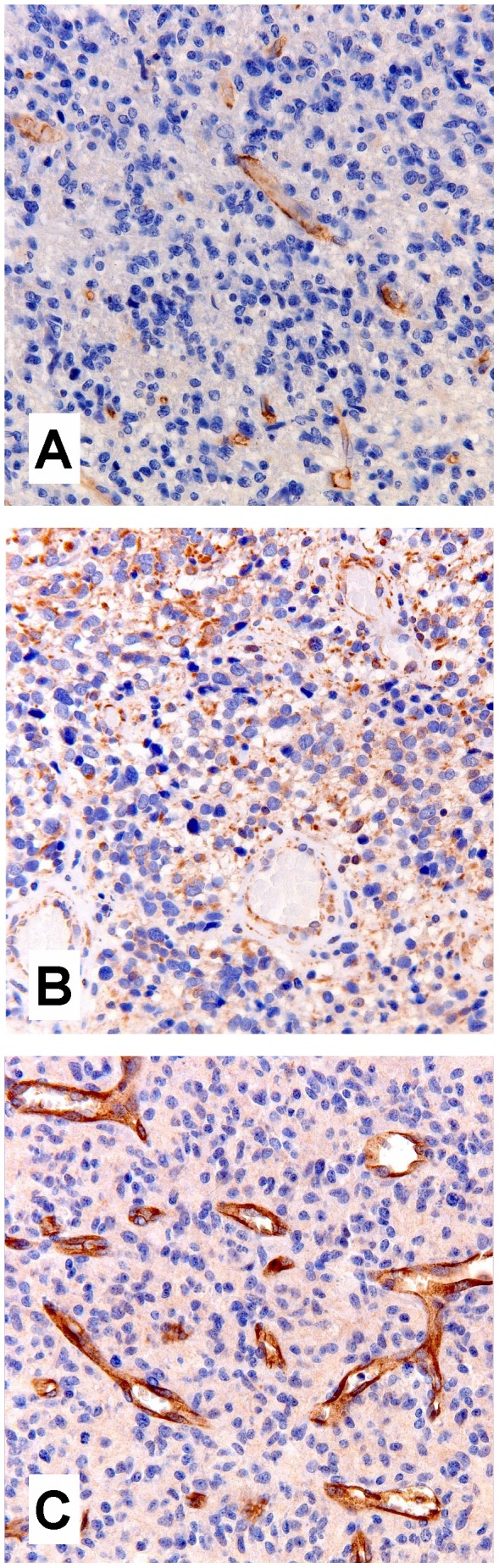
Immunohistochemical staining of ABC-transporters in pHGG sections. Expression of P-gp (A) and BCRP1 (C) is located to the endothelial cells of the tumor vasculature. Whereas MRP1 (B) expression is visualized mainly in the cytoplasm of tumor cells as well as in the vasculature.

To further explore the role of the various ABC transporters in pHGG, and to assess their presence in the (peri)tumoral vasculature, immunohistochemistry was performed on tumor tissue sections of the patients corresponding to the cell cultures (Figure 3). A semi-quantitative analysis of the sections was performed to distinguish between tumor cells and endothelial cells of the BBB (Table 4). P-gp was absent in the glioma cell membranes in most tumor sections, but showed a moderate expression in the tumor vasculature in half of the patients. MRP1 was expressed in both the glioma cells, and the tumor vasculature in most sections. Staining of BCRP1 in glioma cells was mostly negative or weak, while the microvasculature showed intense staining in the majority of the sections. Representative pictures are given in Figure 3.

**Table 4:** Immunohistochemistry scores of P-gp, Mrp1, and Bcrp1 in tumor tissue sections.

	ABCB1/P-gp		ABCC1/MRP1		ABCG2/BCRP1	
	Tumor cells	Vasculature	Tumor cells	Vasculature	Tumor cells	Vasculature
**VUMC-HGG-01**	negative	weak	moderate	moderate	negative	strong
**VUMC-HGG-02**	negative	moderate	weak	moderate	weak	strong
**VUMC-HGG-03**	negative	negative	moderate	moderate	negative	weak
**VUMC-HGG-05**	negative	negative	weak	weak	negative	weak
**VUMC-HGG-06**	weak	negative	negative	negative	negative	strong
**VUMC-HGG-07**	negative	moderate	moderate	moderate	negative	strong
**VUMC-DIPG-01**	negative	moderate	weak	moderate	weak	strong
**VUMC-DIPG-A**	negative	moderate	moderate	strong	negative	strong
**VUMC-DIPG-B**	−	−	−	−	−	−

Protein expression was scored for quantity (percentage of cells, ranging from 0 = 0, 1 = <20%, 2 = 20–40%, 3 = 40–60%, 4 = 60–80%, to 5 = 80–100%), and intensity (0 = absent, 1 = low, 2 = moderate, 3 = high). Using these data, an immunohistochemistry score (IHS) was calculated by multiplying the quantity and staining intensity scores. IHS: 0 = negative, 1–3 = weak, 4–9 = moderate, 10–15 = strong.

## Discussion

Here we show that primary cultures derived from pediatric glioma patients, are sensitive to a number of traditional chemotherapeutics as well as to novel, targeted therapeutics. Moreover, - to our knowledge - we provide the first data on drug sensitivity screening of primary DIPG cultures, suggesting that DIPG *per se* is not necessarily resistant to chemotherapy.

Although there is a substantial heterogeneity in tumor origin and genetic aberrations, a number of drugs show high cytotoxicity in most cell cultures. Generally, the classical, active chemotherapeutics can be divided into two classes: topoisomerase II inhibitors (mitoxantrone, doxorubicin, and etoposide), and alkylating agents (melphalan, BCNU, carboplatin, and thiotepa). We found that melphalan was the only drug to induce significant cell death in all primary cultures, even in the most resistant glioma culture, which was a giant cell glioblastoma. However, the efficacy of melphalan in pediatric gliomas has not been assessed so it is unknown whether sufficient drug levels can be reached locally in these children. Testing in *in vivo* glioma models will show whether melphalan has the potential to produce toxicity at levels that can be attained *in situ*. BCNU, another alkylating agent, also demonstrated high cytotoxicity in most of our pediatric glioma cultures. However, like melphalan, BCNU is considered ineffective in brain tumors, due to its inability to pass the blood-brain barrier. There is some evidence that BCNU-impregnated wafers result in improved survival in adults [13], [14]and a case of successful glioblastoma treatment using BCNU wafers in a child has been described [15], suggesting that this might be a potential drug in the treatment of pHGG when delivered locally. For the treatment of DIPG the use of local delivery, e.g. by convection-enhanced-delivery (CED), with BCNU may be an option.

In contrast to the classical chemotherapeutic agents, the targeted drugs did not prove very effective in our experiments, although the tyrosine kinase inhibitors bosutinib (Src kinase), dasatinib (BCR/ABL, Src kinase) and sorafenib (VEGFR, PDGFR, Raf kinase) showed efficacy in some of our glioma cultures. However, we have to keep in mind that the choice of these compounds for our small molecule screen was based on mRNA expression levels reported previously for pHGG and DIPG, but we did not analyze the expression of the targeted proteins in our cells in detail [16]. Thus, these drugs may prove more effective when targeted agents are chosen based on individualized tissue analysis and mutated targets, as also shown in a clinical setting [17]. Besides, as it appears that multiple aberrant signaling pathways are involved in gliomas, an effective approach presumably requires combined targeted regimens [18]. Since bortezomib induced cell death in most of our cultures in physiologically relevant doses, therapeutic efficacy may increase by including such proteasome inhibitors in a multi-targeted approach. [19]. Indeed, a recent phase-1 clinical trial in adults with recurrent malignant glioma reported some clinical activity of bortezomib [20]. Importantly however, pediatric and adult HGGs are two distinct diseases at the molecular level[21]–[23], which may explain the difference in responses to cytotoxic drugs, of which temozolomide (TMZ) is an illustrative example. TMZ is one of the most commonly used therapeutics for adults with proven efficacy in newly diagnosed and recurrent or progressive gliomas [24], but has not resulted in a better outcome in children with HGG [25], nor DIPG [26]. In fact, also in our primary glioma cultures, temozolomide treatment was ineffective, even when combined with irradiation.

In order to elucidate the differences in effect of drug treatment observed in glioma cultures *in vitro*, and the clinical responses reported in literature, we investigated the role of drug efflux transporters that belong to the ATP-binding cassette (ABC) superfamily. More precisely, we determined the expression of three major drug efflux transporters present in brain: P-glycoprotein/MDR1 (P-gp), Multidrug Resistant Protein 1 (MRP1), and Breast Cancer Resistance Protein 1 (BCRP1), on both the glioma cells and surrounding tissue. P-gp, encoded by the ABCB1 gene, reduces intracellular drug accumulation by acting as an active ATP-driven transmembrane drug transporter [27], and is thought to prevent toxic substances from entering the blood brain barrier (BBB) by its expression in endothelial cells [28]. Using immunohistochemical staining, we show that P-gp is expressed on a large number of endothelial cells of the tumor vasculature in half of the pHGG cultures tested, comparable to the expression in normal brain [29]. No expression of P-gp was detected in glioma cells. These results were confirmed by Western blotting, which showed a complete absence of P-gp protein in primary pHGG cultures. Similar results have been described for adult HGG, where no P-gp was detected in primary adult HGG cultures [30]. Interestingly, however, cell lines derived from human adult glioma generally contain P-gp activity, and although ABCB1 gene expression has been reported in adult HGG [31], this was not always confirmed by immunohistochemical staining [32]. Together, these results suggest that the role of P-gp in pediatric glioma patients is limited to the BBB, and that an anti-tumor effect of therapeutics that are substrate for P-gp is not ruled out when these barriers are bypassed by intratumoral administration. Indeed, a significant effect of doxorubicin, the most important substrate for P-gp, is observed in our drug screen.

Breast cancer resistance protein 1 (BCRP1), encoded by the ABCG2 gene, is a relatively recently discovered drug resistance protein that has been detected in a variety of solid tumors [33]. However, the significance of this protein in human gliomas is unclear. At the predicted molecular weight, BCRP1 was only detected in one of our pediatric glioma cultures, although additional bands of higher molecular weight were detected in half of the cultures, suggesting the formation of oligomers [34]. As a half-transporter, BCRP1 dimerizes to form a functional ABC protein, with two ATP-binding domains and two sets of transmembrane regions. Therefore, these high-order oligomers are likely to be non-functional. The absence of BCRP1 on glioma cells that was observed on Western blot, corresponded with the immunohistochemistry results. In all patient samples BCRP1 is intensely expressed in the endothelial cells of the tumor vasculature, as also seen in endothelial cells of normal brain [29], but hardly on the tumor cells themselves.

Multidrug resistant protein 1 (MRP1), encoded by the ABCC1 gene, is suggested to contribute to the chemoresistance of adult human gliomas. In contrast to P-gp, MRP1 decreases intracellular drug accumulation preferentially by unidirectional, ATP-driven export of toxic agents [35]. Our results indicate that there is also a significant role for MRP1 in drug resistance in pediatric high grade glioma. MRP1 protein expression is displayed in most glioma cultures, independently of the histological type and tumor grade. Of note, there are some discrepancies in the detection of MRP1 on Western blot and IHC (most notably in VUMC-HGG-02) that may be explained by tumor heterogeneity. More importantly, however, we could not relate the expression of MRP1 by the cultures to a resistance pattern to substrates of MRP1 in the drug screen. In order to quantify the role of MRP1 in tumor cell resistance mechanisms, a functional inhibitory assay should be performed, preferably using MRP1 knockout mice. In the tumor sections, MRP1 was also visualized in the endothelial cells in all tumors, suggesting that MRP1 could limit the permeability of its substrates, not only in tumor cells but also across tumoral microvessels. Based on these results, it is not likely that substrates for MRP1 will target pediatric glioma in a satisfying manner, even when using local delivery, unless MRP1 function is inhibited.

In conclusion, all three major ABC transporters are active at the BBB, impeding the delivery of chemotherapeutics to the tumor site. There are higher levels of P-gp and BCRP1 in the endothelial cells of the tumor vasculature than in the glioma cells themselves, which is in line with findings in adult glioma [36], [37]. Targeting P-gp and BCRP1 in the vasculature might therefore enhance the clinical response to chemotherapeutics by inhibiting barrier function, but has a risk of conveying more toxicity, as both P-gp and BCRP are present in comparable levels in normal brain [29]. Since MRP1 is not only present at the BBB, but also seems to be expressed by glioma cells, this could be an even better clinical target. However, it should be stated that the field of drug resistance is very complex, and functional assays and *in vivo* studies are required to assess the role of different drug efflux transporters in relation to glioma drug resistance.

Most inhibitors of ABC transporter activity developed thus far have considerable toxic side effects, and therefore have failed to significantly improve chemotherapeutic efficacy in patients [38]. Interestingly, tyrosine kinase inhibitors (TKIs), which are considered among the most promising agents for HGGs, were reported to antagonize ABC-transporter-related drug resistance[39]–[41]. Therefore, combinations of TKIs, with established chemotherapeutic agents, could hold promise in overcoming drug resistance, although single-agent TKIs did not show much anti-tumor activity in our screen.

Potential limitations of the study are the limited sample size, and the design of the customized drug screen. The drug concentrations used in the present study were mainly based on information obtained in adult glioma cell lines, which could bias our results in both directions. The purpose of our screen was to identify potential effective anti-glioma therapeutics, rather than to exclude therapeutics for future protocols. Therefore, it is important to emphasize that therapeutics that were not considered effective in this experimental design, should not necessarily be considered ineffective in pediatric glioma without further exploration, as higher doses might prove successful. When aiming for translation to the clinical situation, *in vivo* experiments with HGG and DIPG xenograftmodels will be crucial to further elucidate the role of the drug efflux transporters in relation to drug resistance.

As some of the drugs that display a high toxicity in cultures were never found to be beneficial in clinical trials, the understanding of drug resistance in pHGG is essential for the development of efficient therapies. Our data suggest that the presence of drug efflux pumps in the blood-tumor barrier may constitute an important first line of resistance of pHGG to such treatment, and the efficacy may be further impaired by expression of a subset of these, as well as other drug resistance proteins in the glioma cells themselves. Possible approaches to overcome these mechanisms of resistances are direct intratumoral drug delivery, such as convection enhanced delivery (CED), and the use of drugs that are not substrate for these drug transporters.

## Materials and Methods

### Processing of Tumor Material and Cell Culture

Single cell cultures were established from biopsy samples derived from pediatric glioblastoma multiforme, anaplastic astrocytoma, anaplastic oligodendroglioma and diffuse intrinsic pontine glioma (DIPG) or from DIPG autopsy samples. Informed consent was obtained according to institutionally-approved protocols. Tumor pieces were collected into DMEM (Dulbecco’s Modified Eagles Medium, PAA Laboratories GmbH, Pasching, Austria) and washed twice with PBS to remove blood clots. Samples were sliced into small (3–5 mm^3^) pieces and either mechanically dissociated by filtering through a cell strainer (BD Falcon Biosciences, Bedford, USA)), or dissociated by incubation with Accutase (PAA Laboratories GmbH, Pasching, Austria). Single cells were seeded in DMEM-F12, constituted with stable glutamine, 10% fetal bovine serum (PerBio Science Nederland B.V., Etten-Leur, The Netherlands), 1% penicillin/streptomycin (PAA Laboratories GmbH, Pasching, Austria), and 0,5% sodium pyruvate. For primary astrocytes 15% fetal bovine serum was used. Cells were grown at 37°C in a 5% CO_2_ humified atmosphere.

### Karyotyping and Comparative Genomic Hybridization (CGH)

Malignancy of the established cell lines was confirmed by determining the chromosomal aberrations, either by classical karyotyping, or by array CGH. For cytogenetic analysis, cells were harvested according to standard cytogenetic techniques. Briefly, cells were treated with demecolcine (final concentration 0.4 µg/ml; Sigma) for 2 hours, trypsinized, treated with a 75 mM KCl hypotonic solution and finally fixed using methanol:acetic acid (3∶1). Metaphase spreads were prepared by dropping the fixed cells on glass slides. Karyotyping was performed on GTG banded metaphase cells. Metaphase spreads were observed using a Zeiss Axioskop 20 microscope. Images were captured using a Cytovision imaging system (Leica-microsystems, United Kingdom). For array CGH, genomic DNA was isolated using the Wizard genomic DNA purification kit (Promega) and hybridized to NimbleGen Human CGH 3×720 K Whole-Genome Tiling v2.0 Arrays (Roche Diagnostics) according to the manufacturer’s instructions. Data analyses were performed using the Nexus 5.0 software.

### Drug Screen

The primary pediatric glioma- and astrocyte cultures were exposed to a customized chemical screen, consisting of 21 chemotherapeutic drugs, at dose ranges that were based on published *in vitro* IC_50_s in malignant glioma (Table 2 and 3). Fifteen hundred cells were seeded per well in 96-well tissue culture plates. Cells in each well were treated with a different compound at two different concentrations. For treatment with temozolomide and olaparib, treatment was done with paired 96-well plates, where one plate was exposed to 3 Gray in a Gammacell® 220 Research Irradiator (MDS Nordion, Canada) after 24 hours of incubation, and the other plate was a non-irradiated control. After 96 hours cell survival was assessed using the Acumen ^e^X3 laser cytometer (TTO Labtech, UK), using 300 mM of 49,6-diamidino-2-phenylindole dihydrochloride (DAPI) (Sigma-Aldrich) as readout. Results were analyzed using Acumen Explorer software, calculating the survival percentage for each compound tested in the assay. Each experiment was performed at least four times. A coëfficient of variation (CV) and Z’ factor were calculated to assess the reproducibility and robustness of the small molecule screens, where CV = SD/µ and Z’ = 1−(3σ_c+_+3σ_c−_)/Iµ_c+_−µ_c−_I [42].

### Cell Extraction and Western Blotting

Cell cultures were washed twice in PBS, and cell-free extracts were made by resuspension in lysis buffer (50% Tris/HCl pH7.6, 25% MiliQ, 20% glycerol, 4% Protease Inhibitor Cocktail, 0,5% 1 M Dithiotreitol (DTT), 0,5% NP-40) and sonification. The samples were then centrifuged at 13000 rpm for 10 minutes at 4°C, and the supernatants were subsequently used for Western blotting. Equal amounts of protein were loaded on a 4–12% gradient SDS polyacrylamide gel and transferred to PVDF membranes (Millipore, Amsterdam, The Netherlands). The membranes were washed, blocked with 5% milk, and incubated overnight with an 1∶50 diluted antibody against P-gp/ABCB1 (MDR1/clone JSB-1 mab4120, Chemicon, Millipore, Temecula, California), BCRP/ABCG2 (BXP-21 Ab3380, Abcam, Cambridge, United Kingdom), MRP1/ABCC1 (ABCC1 A10 HPA002380, Sigma-Aldrich), or with an 1∶15,000 diluted antibody directed against β-Actin (Millipore, MAB1501R). After several washes the membranes were incubated with anti-mouse or anti-rabbit IgG HRP (Dako, Glostrup, Denmark) in a dilution of 1∶3,000. Antibody binding was detected using Enhanced Chemiluminescence (GE Healthcare, Buckinghamshire, UK).

### Immunohistochemistry

Paraffin-embedded tissue was sliced into 5 µm sections, deparaffinized and rehydrated by washing in xylene and ethanol series. Endogenous peroxidase activity was blocked with 0.3% hydrogen peroxidase in methanol for 30 minutes. The sections were washed and heated in citrate buffer pH6, and gradually cooled to room temperature. After a PBS wash, the sections that were stained for P-gp were blocked with 5% normal goat serum (NGS) in PBS for thirty minutes prior to primary antibody incubation. Sections were incubated for one hour at room temperature with an 1∶20 diluted antibody against P-gp (MDR1/clone JSB-1 mab4120, Chemicon, Millipore, Temecula, California), an 1∶40 diluted antibody against BCRP1 (BXP-21 Ab3380, Abcam, Cambridge, United Kingdom), or an 1∶75 diluted antibody against MRP1 (ABCC1 A10 HPA002380, Sigma-Aldrich). For the detection of the primary antibody, EnVision anti-mouse/anti-rabbit (ImmunoLogic, Duiven, The Netherlands) was used according the manufacturer’s instructions. Peroxidase activity was detected using 3,3-diaminobenzidine-Tetrachloride (DAB) (Sigma, USA) in 0.1% hydrogen peroxide. All sections were counterstained with haematoxylin. Protein expression was scored for quantity (percentage of cells, ranging from 0 = 0, 1 = <20%, 2 = 20–40%, 3 = 40–60%, 4 = 60–80%, to 5 = 80–100%), and intensity (0 = absent, 1 = low, 2 = moderate, 3 = high). Using these data, an immunohistochemistry score (IHS) was calculated by multiplying the quantity and staining intensity scores. IHS: 0 = negative, 1–3 = weak, 4–9 = moderate, 10–15 = strong. Images were made using a Zeiss Axioskop microscope (HBO100W/Z), equipped with a Canon digital camera (Canon PowerShot A460, Canon Inc., Tokyo, Japan); imaging software is Canon Utilities, ZoomBrowser Ex. 5.7, Canon Inc, Nort Ryde, Australia.

## Supporting Information

Figure S1 ***In silico*** **analysis.** *In silico* analysis of mRNA expression using R2 analysis software on datasets of non-malignant brain tissue (light blue), versus datasets of pediatric HGG and DIPG (dark blue).
(TIF)

Figure S2 **Cell proliferation assay, as determined by BrdU incorporation.** Immunofluorescence showing the percentages of BrdU incorporation (green) as compared to DAPI staining (blue) in VUMC-HGG-01, VUMC-HGG-05, and VUMC-DIPG-A.
(TIF)

Material and Methods S1 **Supplementary Material and Methods.**
(DOCX)

## References

[1] Broniscer A, Gajjar A (2004) Supratentorial high-grade astrocytoma and diffuse brainstem glioma: two challenges for the pediatric oncologist. Oncologist 9: 197–206. 15047924 10.1634/theoncologist.9-2-197

[2] Tamber MS, Rutka JT (2003) Pediatric supratentorial high-grade gliomas. Neurosurg Focus 14: e1. 10.3171/foc.2003.14.2.2 15727422

[3] Jansen MHA, van Vuurden DG, Vandertop WP, Kaspers GJL (2012) Diffuse intrinsic pontine gliomas: a systematic update on clinical trials and biology. Cancer Treat Rev 38: 27–35. 21764221 10.1016/j.ctrv.2011.06.007

[4] Hargrave D, Bartels U, Bouffet E (2006) Diffuse brainstem glioma in children: critical review of clinical trials. Lancet Oncol 7: 241–248. 16510333 10.1016/S1470-2045(06)70615-5

[5] Broniscer A (2006) Past, present, and future strategies in the treatment of high-grade glioma in children. Cancer Invest 24: 77–81. 16466996 10.1080/07357900500449702

[6] Ueno M, Nakagawa T, Wu B, Onodera M, Huang CL, et al (2010) Transporters in the brain endothelial barrier. Curr Med Chem 17: 1125–1138. 20175745 10.2174/092986710790827816

[7] Bredel M (2001) Anticancer drug resistance in primary human brain tumors. Brain Res Brain Res Rev 35: 161–204. 11336781 10.1016/s0165-0173(01)00045-5

[8] Nies AT (2007) The role of membrane transporters in drug delivery to brain tumors. Cancer Lett 254: 11–29. 17275180 10.1016/j.canlet.2006.12.023

[9] Decleves X, Amiel A, Delattre JY, Scherrmann JM (2006) Role of ABC transporters in the chemoresistance of human gliomas. Curr Cancer Drug Targets 6: 433–445. 16918310 10.2174/156800906777723930

[10] Doyle LA, Yang W, Abruzzo LV, Krogmann T, Gao Y, et al (1998) A multidrug resistance transporter from human MCF-7 breast cancer cells. Proc Natl Acad Sci U S A 95: 15665–15670. 9861027 10.1073/pnas.95.26.15665 PMC28101

[11] Whelan RD, Waring CJ, Wolf CR, Hayes JD, Hosking LK, et al (1992) Over-expression of P-glycoprotein and glutathione S-transferase pi in MCF-7 cells selected for vincristine resistance in vitro. Int J Cancer 52: 241–246. 1355756 10.1002/ijc.2910520215

[12] Hooijberg JH, Peters GJ, Assaraf YG, Kathmann I, Priest DG, et al (2003) The role of multidrug resistance proteins MRP1, MRP2 and MRP3 in cellular folate homeostasis. Biochem Pharmacol 65: 765–771. 12628490 10.1016/s0006-2952(02)01615-5

[13] Dixit S, Hingorani M, Achawal S, Scott I (2011) The sequential use of carmustine wafers (Gliadel(R)) and post-operative radiotherapy with concomitant temozolomide followed by adjuvant temozolomide: a clinical review. Br J Neurosurg 25: 459–469. 21344976 10.3109/02688697.2010.550342

[14] Hart MG, Grant R, Garside R, Rogers G, Somerville M, et al.. (2011) Chemotherapy wafers for high grade glioma. Cochrane Database Syst Rev CD007294. 10.1002/14651858.CD007294.pub2 PMC6457755 21412902

[15] Marquez-Rivas J, Ramirez G, Ollero-Ortiz A, Gimenez-Pando J, Emmerich J, et al (2010) Initial experience involving treatment and retreatment with carmustine wafers in combination with oral temozolomide: long-term survival in a child with relapsed glioblastoma multiforme. J Pediatr Hematol Oncol 32: e202–e206. 20523247 10.1097/MPH.0b013e3181e0d16b

[16] Paugh BS, Qu C, Jones C, Liu Z, Damowicz-Brice M, et al (2010) Integrated molecular genetic profiling of pediatric high-grade gliomas reveals key differences with the adult disease. J Clin Oncol 28: 3061–3068. 20479398 10.1200/JCO.2009.26.7252 PMC2903336

[17] Wolff J, Brown R, Buryanek J, Pfister S, Vats T, et al.. (2011) Preliminary experience with personalized and targeted therapy for pediatric brain tumors. Pediatr Blood Cancer. 10.1002/pbc.23402 22162424

[18] Stommel JM, Kimmelman AC, Ying H, Nabioullin R, Ponugoti AH, et al (2007) Coactivation of receptor tyrosine kinases affects the response of tumor cells to targeted therapies. Science 318: 287–290. 17872411 10.1126/science.1142946

[19] Horton TM, Pati D, Plon SE, Thompson PA, Bomgaars LR, et al (2007) A phase 1 study of the proteasome inhibitor bortezomib in pediatric patients with refractory leukemia: a Children's Oncology Group study. Clin Cancer Res 13: 1516–1522. 17332297 10.1158/1078-0432.CCR-06-2173

[20] Phuphanich S, Supko JG, Carson KA, Grossman SA, Burt Nabors L, et al (2010) Phase 1 clinical trial of bortezomib in adults with recurrent malignant glioma. J Neurooncol 100: 95–103. 20213332 10.1007/s11060-010-0143-7 PMC3811025

[21] Suri V, Das P, Pathak P, Jain A, Sharma MC, et al (2009) Pediatric glioblastomas: a histopathological and molecular genetic study. Neuro Oncol 11: 274–280. 18981259 10.1215/15228517-2008-092 PMC2718971

[22] Qu HQ, Jacob K, Fatet S, Ge B, Barnett D, et al (2010) Genome-wide profiling using single-nucleotide polymorphism arrays identifies novel chromosomal imbalances in pediatric glioblastomas. Neuro Oncol 12: 153–163. 20150382 10.1093/neuonc/nop001 PMC2940568

[23] Paugh BS, Qu C, Jones C, Liu Z, Damowicz-Brice M, et al (2010) Integrated molecular genetic profiling of pediatric high-grade gliomas reveals key differences with the adult disease. J Clin Oncol 28: 3061–3068. 20479398 10.1200/JCO.2009.26.7252 PMC2903336

[24] Stupp R, Hegi ME, Mason WP, van den Bent MJ, Taphoorn MJB, et al (2009) Effects of radiotherapy with concomitant and adjuvant temozolomide versus radiotherapy alone on survival in glioblastoma in a randomised phase III study: 5-year analysis of the EORTC-NCIC trial. Lancet Oncol 10: 459–466. 19269895 10.1016/S1470-2045(09)70025-7

[25] Cohen KJ, Pollack IF, Zhou T, Buxton A, Holmes EJ, et al (2011) Temozolomide in the treatment of high-grade gliomas in children: a report from the Children's Oncology Group. Neuro Oncol 13: 317–323. 21339192 10.1093/neuonc/noq191 PMC3064602

[26] Cohen KJ, Heideman RL, Zhou T, Holmes EJ, Lavey RS, et al (2011) Temozolomide in the treatment of children with newly diagnosed diffuse intrinsic pontine gliomas: a report from the Children's Oncology Group. Neuro Oncol 13: 410–416. 21345842 10.1093/neuonc/noq205 PMC3064697

[27] Bredel M, Zentner J (2002) Brain-tumour drug resistance: the bare essentials. Lancet Oncol 3: 397–406. 12142169 10.1016/s1470-2045(02)00786-6

[28] Cordon-Cardo C, O'Brien JP, Casals D, Rittman-Grauer L, Biedler JL, et al (1989) Multidrug-resistance gene (P-glycoprotein) is expressed by endothelial cells at blood-brain barrier sites. Proc Natl Acad Sci U S A 86: 695–698. 2563168 10.1073/pnas.86.2.695 PMC286540

[29] Daood M, Tsai C, Hdab-Barmada M, Watchko JF (2008) ABC transporter (P-gp/ABCB1, MRP1/ABCC1, BCRP/ABCG2) expression in the developing human CNS. Neuropediatrics 39: 211–218. 19165709 10.1055/s-0028-1103272 PMC2821654

[30] Bahr O, Rieger J, Duffner F, Meyermann R, Weller M, et al (2003) P-glycoprotein and multidrug resistance-associated protein mediate specific patterns of multidrug resistance in malignant glioma cell lines, but not in primary glioma cells. Brain Pathol 13: 482–494. 14655754 10.1111/j.1750-3639.2003.tb00479.x PMC8095903

[31] Kirches E, Oda Y, Von Bossanyi P, Diete S, Schneider T, et al (1997) Mdr1 mRNA expression differs between grade III astrocytomas and glioblastomas. Clin Neuropathol 16: 34–36. 9020393

[32] Henson JW, Cordon-Cardo C, Posner JB (1992) P-glycoprotein expression in brain tumors. J Neurooncol 14: 37–43. 1361524 10.1007/BF00170943

[33] Ni Z, Bikadi Z, Rosenberg MF, Mao Q (2010) Structure and function of the human breast cancer resistance protein (BCRP/ABCG2). Curr Drug Metab 11: 603–617. 20812902 10.2174/138920010792927325 PMC2950214

[34] Litman T, Jensen U, Hansen A, Covitz KM, Zhan Z, et al (2002) Use of peptide antibodies to probe for the mitoxantrone resistance-associated protein MXR/BCRP/ABCP/ABCG2. Biochim Biophys Acta 1565: 6–16. 12225847 10.1016/s0005-2736(02)00492-3

[35] Bredel M (2001) Anticancer drug resistance in primary human brain tumors. Brain Res Brain Res Rev 35: 161–204. 11336781 10.1016/s0165-0173(01)00045-5

[36] Henson JW, Cordon-Cardo C, Posner JB (1992) P-glycoprotein expression in brain tumors. J Neurooncol 14: 37–43. 1361524 10.1007/BF00170943

[37] Sawada T, Kato Y, Sakayori N, Takekawa Y, Kobayashi M (1999) Expression of the multidrug-resistance P-glycoprotein (Pgp, MDR-1) by endothelial cells of the neovasculature in central nervous system tumors. Brain Tumor Pathol 16: 23–27. 10532420 10.1007/BF02478898

[38] Shukla S, Wu CP, Ambudkar SV (2008) Development of inhibitors of ATP-binding cassette drug transporters: present status and challenges. Expert Opin Drug Metab Toxicol 4: 205–223. 18248313 10.1517/17425255.4.2.205

[39] Azzariti A, Porcelli L, Simone GM, Quatrale AE, Colabufo NA, et al (2010) Tyrosine kinase inhibitors and multidrug resistance proteins: interactions and biological consequences. Cancer Chemother Pharmacol 65: 335–346. 19495754 10.1007/s00280-009-1039-0

[40] Shi Z, Peng XX, Kim IW, Shukla S, Si QS, et al (2007) Erlotinib (Tarceva, OSI-774) antagonizes ATP-binding cassette subfamily B member 1 and ATP-binding cassette subfamily G member 2-mediated drug resistance. Cancer Res 67: 11012–11020. 18006847 10.1158/0008-5472.CAN-07-2686

[41] Mi Yj, Liang Yj, Huang HB, Zhao HY, Wu CP, et al (2010) Apatinib (YN968D1) reverses multidrug resistance by inhibiting the efflux function of multiple ATP-binding cassette transporters. Cancer Res 70: 7981–7991. 20876799 10.1158/0008-5472.CAN-10-0111 PMC2969180

[42] Zhang J, Chung T, Oldenburg K (1999) A Simple Statistical Parameter for Use in Evaluation and Validation of High Throughput Screening Assays. J Biomol Screen 4: 67–73. 10838414 10.1177/108705719900400206

[43] Fruehauf JP, Brem H, Brem S, Sloan A, Barger G, et al (2006) In vitro drug response and molecular markers associated with drug resistance in malignant gliomas. Clin Cancer Res 12: 4523–4532. 16899598 10.1158/1078-0432.CCR-05-1830

[44] Wolff JE, Trilling T, Molenkamp G, Egeler RM, Jurgens H (1999) Chemosensitivity of glioma cells in vitro: a meta analysis. J Cancer Res Clin Oncol 125: 481–486. 10480340 10.1007/s004320050305 PMC12172386

[45] Ng WH, Wan GQ, Too HP (2007) Higher glioblastoma tumour burden reduces efficacy of chemotherapeutic agents: in vitro evidence. J Clin Neurosci 14: 261–266. 17258135 10.1016/j.jocn.2005.11.010

[46] Wolff JE, Trilling T, Molenkamp G, Egeler RM, Jurgens H (1999) Chemosensitivity of glioma cells in vitro: a meta analysis. J Cancer Res Clin Oncol 125: 481–486. 10480340 10.1007/s004320050305 PMC12172386

[47] Riccardi R, Riccardi A, Lasorella A, Di Rocco C, Carelli G, et al (1994) Clinical pharmacokinetics of carboplatin in children. Cancer Chemother Pharmacol 33: 477–483. 8137457 10.1007/BF00686504

[48] Wolff JE, Trilling T, Molenkamp G, Egeler RM, Jurgens H (1999) Chemosensitivity of glioma cells in vitro: a meta analysis. J Cancer Res Clin Oncol 125: 481–486. 10480340 10.1007/s004320050305 PMC12172386

[49] Hempel G, Flege S, Wurthwein G, Boos J (2002) Peak plasma concentrations of doxorubicin in children with acute lymphoblastic leukemia or non-Hodgkin lymphoma. Cancer Chemother Pharmacol 49: 133–141. 11862427 10.1007/s00280-001-0392-4

[50] Fruehauf JP, Brem H, Brem S, Sloan A, Barger G, et al (2006) In vitro drug response and molecular markers associated with drug resistance in malignant gliomas. Clin Cancer Res 12: 4523–4532. 16899598 10.1158/1078-0432.CCR-05-1830

[51] Kato Y, Nishimura Si, Sakura N, Ueda K (2003) Pharmacokinetics of etoposide with intravenous drug administration in children and adolescents. Pediatr Int 45: 74–79. 12654074 10.1046/j.1442-200x.2003.01675.x

[52] Souid AK, Dubowy RL, Blaney SM, Hershon L, Sullivan J, et al (2003) Phase I clinical and pharmacologic study of weekly cisplatin and irinotecan combined with amifostine for refractory solid tumors. Clin Cancer Res 9: 703–710. 12576438

[53] Kupczyk-Subotkowska L, Siahaan TJ, Basile AS, Friedman HS, Higgins PE, et al (1997) Modulation of melphalan resistance in glioma cells with a peripheral benzodiazepine receptor ligand-melphalan conjugate. J Med Chem 40: 1726–1730. 9171882 10.1021/jm960592p

[54] Wolff JE, Trilling T, Molenkamp G, Egeler RM, Jurgens H (1999) Chemosensitivity of glioma cells in vitro: a meta analysis. J Cancer Res Clin Oncol 125: 481–486. 10480340 10.1007/s004320050305 PMC12172386

[55] Jiang X, Xin H, Sha X, Gu J, Jiang Y, et al (2011) PEGylated poly(trimethylene carbonate) nanoparticles loaded with paclitaxel for the treatment of advanced glioma: in vitro and in vivo evaluation. Int J Pharm 420: 385–394. 21920419 10.1016/j.ijpharm.2011.08.052

[56] Doz F, Gentet JC, Pein F, Frappaz D, Chastagner P, et al (2001) Phase I trial and pharmacological study of a 3-hour paclitaxel infusion in children with refractory solid tumours: a SFOP study. Br J Cancer 84: 604–610. 11237379 10.1054/bjoc.2000.1637 PMC2363793

[57] Fruehauf JP, Brem H, Brem S, Sloan A, Barger G, et al (2006) In vitro drug response and molecular markers associated with drug resistance in malignant gliomas. Clin Cancer Res 12: 4523–4532. 16899598 10.1158/1078-0432.CCR-05-1830

[58] Sankar A, Thomas DG, Darling JL (1999) Sensitivity of short-term cultures derived from human malignant glioma to the anti-cancer drug temozolomide. Anticancer Drugs 10: 179–185. 10211548 10.1097/00001813-199902000-00006

[59] Estlin EJ, Lashford L, Ablett S, Price L, Gowing R, et al (1998) Phase I study of temozolomide in paediatric patients with advanced cancer. United Kingdom Children's Cancer Study Group. Br J Cancer 78: 652–661. 9744506 10.1038/bjc.1998.555 PMC2063055

[60] Kletzel M, Kearns GL, Wells TG, Thompson HCJ (1992) Pharmacokinetics of high dose thiotepa in children undergoing autologous bone marrow transplantation. Bone Marrow Transplant 10: 171–175. 1525606

[61] Wolff JE, Trilling T, Molenkamp G, Egeler RM, Jurgens H (1999) Chemosensitivity of glioma cells in vitro: a meta analysis. J Cancer Res Clin Oncol 125: 481–486. 10480340 10.1007/s004320050305 PMC12172386

[62] Moore AS, Norris R, Price G, Nguyen T, Ni M, et al (2011) Vincristine pharmacodynamics and pharmacogenetics in children with cancer: A limited-sampling, population modelling approach. J Paediatr Child Health 47: 875–882. 21658147 10.1111/j.1440-1754.2011.02103.x

[63] Styczynski J, Olszewska-Slonina D, Kolodziej B, Napieraj M, Wysocki M (2006) Activity of bortezomib in glioblastoma. Anticancer Res 26: 4499–4503. 17201170

[64] Coluccia AML, Benati D, Dekhil H, De Filippo A, Lan C, et al (2006) SKI-606 decreases growth and motility of colorectal cancer cells by preventing pp60(c-Src)-dependent tyrosine phosphorylation of beta-catenin and its nuclear signaling. Cancer Res 66: 2279–2286. 16489032 10.1158/0008-5472.CAN-05-2057

[65] Homsi J, Cubitt CL, Zhang S, Munster PN, Yu H, et al (2009) Src activation in melanoma and Src inhibitors as therapeutic agents in melanoma. Melanoma Res 19: 167–175. 19434004 10.1097/CMR.0b013e328304974c

[66] Premkumar DR, Jane EP, Agostino NR, Scialabba JL, Pollack IF (2010) Dasatinib synergizes with JSI-124 to inhibit growth and migration and induce apoptosis of malignant human glioma cells. J Carcinog 9. 10.4103/1477-3163.65448 PMC2924609 20808823

[67] Wang MY, Lu KV, Zhu S, Dia EQ, Vivanco I, et al (2006) Mammalian target of rapamycin inhibition promotes response to epidermal growth factor receptor kinase inhibitors in PTEN-deficient and PTEN-intact glioblastoma cells. Cancer Res 66: 7864–7869. 16912159 10.1158/0008-5472.CAN-04-4392

[68] Alonso MM, Jiang H, Yokoyama T, Xu J, Bekele NB, et al (2008) Delta-24-RGD in combination with RAD001 induces enhanced anti-glioma effect via autophagic cell death. Mol Ther 16: 487–493. 18253154 10.1038/sj.mt.6300400

[69] van Vuurden D, Hulleman E, Meijer O, Wedekind L, Kool M, et al.. (2011) PARP inhibition sensitizes childhood high grade glioma, medulloblastoma and ependymoma to radiation. Oncotarget. 10.18632/oncotarget.362 PMC3282104 22184287

[70] Hjelmeland MD, Hjelmeland AB, Sathornsumetee S, Reese ED, Herbstreith MH, et al (2004) SB-431542, a small molecule transforming growth factor-beta-receptor antagonist, inhibits human glioma cell line proliferation and motility. Mol Cancer Ther 3: 737–745. 15210860

[71] Jane EP, Premkumar DR, Pollack IF (2006) Coadministration of sorafenib with rottlerin potently inhibits cell proliferation and migration in human malignant glioma cells. J Pharmacol Exp Ther 319: 1070–1080. 16959960 10.1124/jpet.106.108621

[72] Geoerger B, Kerr K, Tang CB, Fung KM, Powell B, et al (2001) Antitumor activity of the rapamycin analog CCI-779 in human primitive neuroectodermal tumor/medulloblastoma models as single agent and in combination chemotherapy. Cancer Res 61: 1527–1532. 11245461

[73] Rich JN, Sathornsumetee S, Keir ST, Kieran MW, Laforme A, et al (2005) ZD6474, a novel tyrosine kinase inhibitor of vascular endothelial growth factor receptor and epidermal growth factor receptor, inhibits tumor growth of multiple nervous system tumors. Clin Cancer Res 11: 8145–8157. 16299247 10.1158/1078-0432.CCR-05-0319

