# Supplementary figures and images for: In Vitro Drug Response and Efflux Transporters Associated with Drug Resistance in Pediatric High Grade Glioma and Diffuse Intrinsic Pontine Glioma
Source: PLoS One. 2013 Apr 29;8(4):e61512. doi: 10.1371/journal.pone.0061512 (PMC3639279; doi:10.1371/journal.pone.0061512)

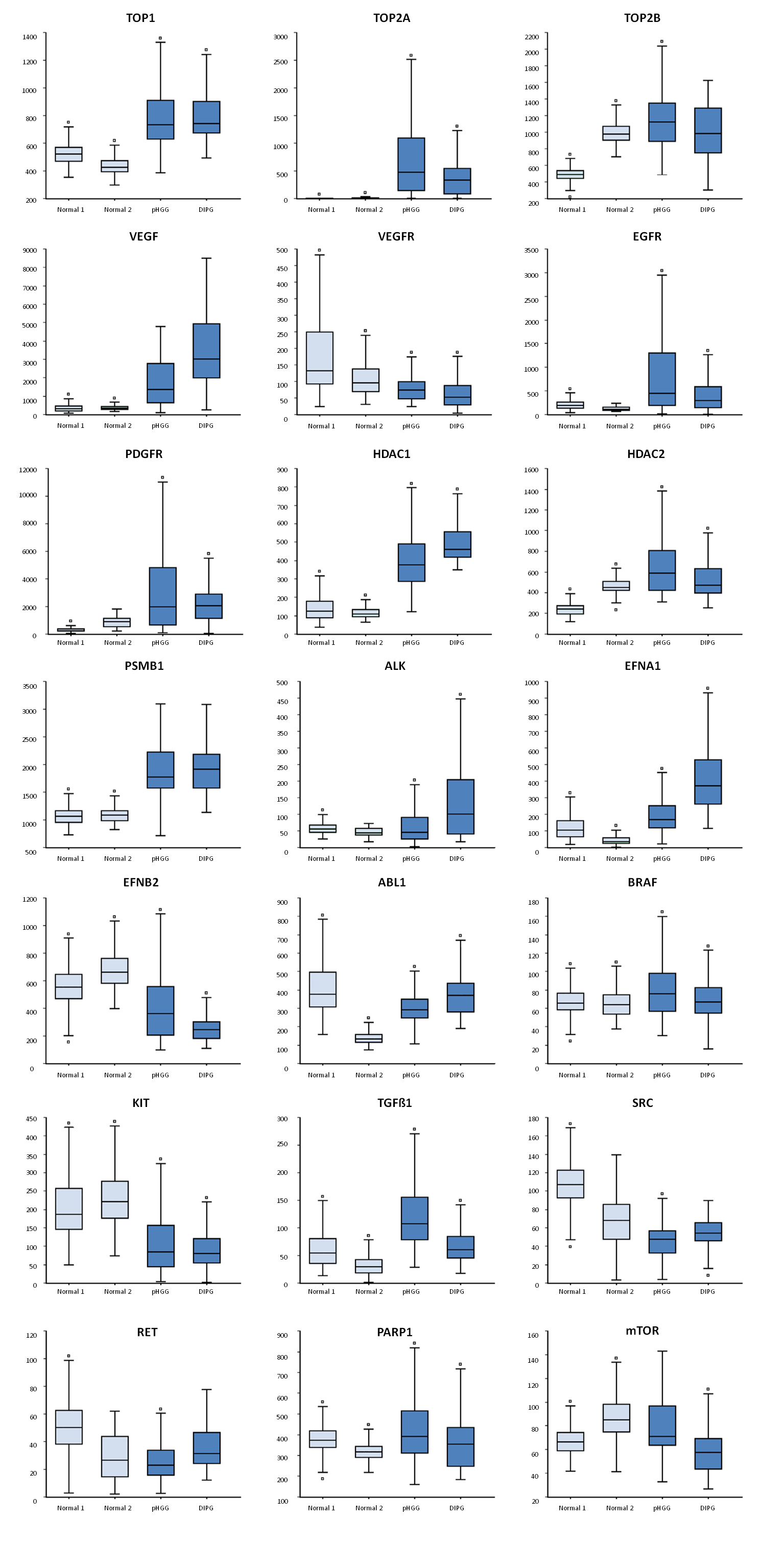

Supplement: Figure S1 — In silico analysis. In silico analysis of mRNA expression using R2 analysis software on datasets of non-malignant brain tissue (light blue), versus datasets of pediatric HGG and DIPG (dark blue). (TIF) [file pone.0061512.s001.tif]

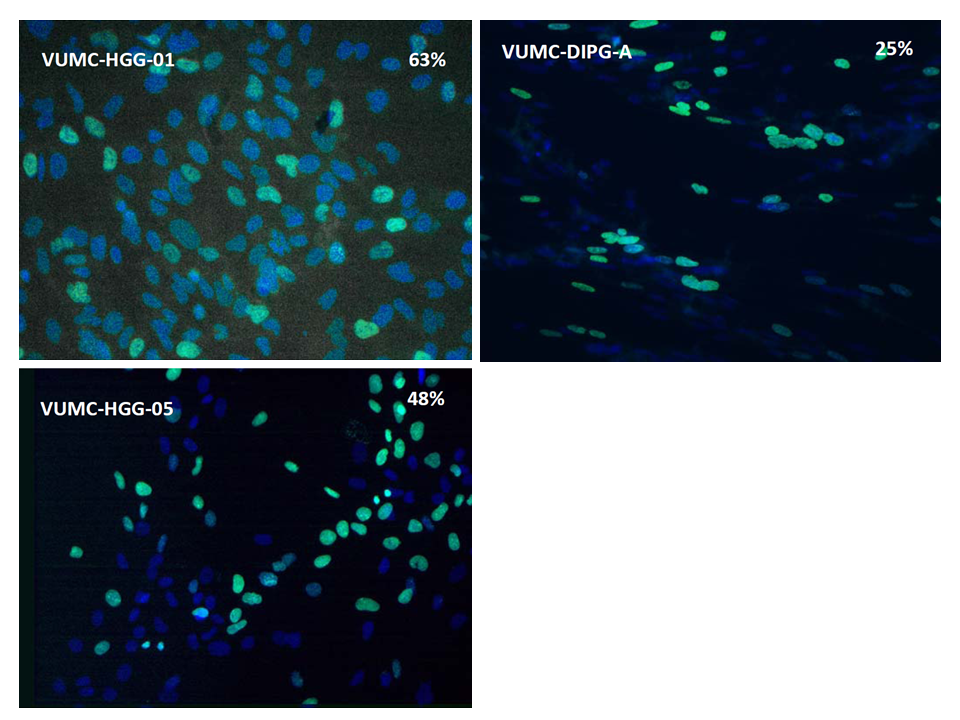

Supplement: Figure S2 — Cell proliferation assay, as determined by BrdU incorporation. Immunofluorescence showing the percentages of BrdU incorporation (green) as compared to DAPI staining (blue) in VUMC-HGG-01, VUMC-HGG-05, and VUMC-DIPG-A. (TIF) [file pone.0061512.s002.tif]
