# Supplementary material for: In Vitro Drug Response and Efflux Transporters Associated with Drug Resistance in Pediatric High Grade Glioma and Diffuse Intrinsic Pontine Glioma
Source: PLoS One. 2013 Apr 29;8(4):e61512. doi: 10.1371/journal.pone.0061512 (PMC3639279; doi:10.1371/journal.pone.0061512)
Supplement: Material and Methods S1 — Supplementary Material and Methods. (DOCX) [file pone.0061512.s003.docx]

Supplementary Material and Methods:

### In silico analysis

R2, a microarray analysis and visualization platform, provided by the Department of Human Genetics of the Academic Medical Centre, Amsterdam, The Netherlands (<http://r2.amc.nl>), was used to obtain an overview of target gene mRNA expression in pediatric high-grade glioma and DIPG. MAS5.0 normalized datasets of childhood HGG and DIPG (n=53; [{"type":"entrez-geo","attrs":{"text":"GSE19578","term_id":"19578"}}GSE19578](http://www.ncbi.nlm.nih.gov/geo/query/acc.cgi?acc=GSE19578))(16) were compared to normal prefrontal cortex (n=44; [{"type":"entrez-geo","attrs":{"text":"GSE13564","term_id":"13564"}}GSE13564](http://www.ncbi.nlm.nih.gov/geo/query/acc.cgi?acc=GSE13564))(75), and normal hippocampus, entorhinal cortex, superior frontal gyrus and potcentral gyrus (n=172; GSE1182)(76).

**BrdU incorporation**

For bromodeoxyuridine (BrdU) incorporation studies, cells were pulsed O/N with 1:100 uM BrdU. Cells were washed in PBS, fixed in 75% EtOH for 20 min and incubated with anti-BrdU antibody (Alexa fluor 488, 1:100), and 4’, 6-diamidino-2-phenylindole (DAPI). Between each incubation step, coverslips were washed in PBS. For quantification, 100 nuclei were counted and correlating BrdU percentages were calculated.
